# Data on localization of coxsackievirus and adenovirus receptor (CAR) in the embryonic rat brain

**DOI:** 10.1016/j.dib.2019.103726

**Published:** 2019-03-07

**Authors:** Mo Chen, Takako Kato, Yukio Kato

**Affiliations:** a Division of Life Science, Graduate School of Agriculture, Meiji University, Kawasaki, Kanagawa, 214-8571, Japan; b Institute for Endocrinology, Meiji University, Kawasaki, Kanagawa, 214-8571, Japan; c Department of the Life Science, School of Agriculture, Meiji University, Kawasaki, Kanagawa, 214-8571, Japan

## Abstract

The data in the present article are related to the previous article entitled “Coxsackievirus and adenovirus receptor-positive cells compose the putative stem/progenitor cell niches in the marginal cell layer and parenchyma of the rat anterior pituitary” (Chen et al., 2013). The data describe the characteristic localization of coxsackievirus and adenovirus receptor (CAR), a junctional adhesion molecule involved in the regulation of cell–cell interactions, migration, proliferation, and growth (Coyne and Bergelson, 2005, Matthaus et al., 2017, Raschperger et al., 2006, Schiestl and Gietz, 1989) and in the stem/progenitor cell niche in the embryonic rat pituitary gland (Chen et al., 2013, Yoshida et al., 2016). Immunohistochemical analyses of CAR showed frequent colocalization with SOX2 in the embryonic rat brain, except for choroid plexus cells. CAR showed distinct apical and basolateral polarity. These data contribute to our understanding of prenatal brain development.

**Table:** Specifications table

Subject area	*Biology*
More specific subject area	*Developmental biology*
Type of data	*Immunohistochemistry*
Data format	*Figures and table*
How data was acquired	*Immunofluorescence images were obtained by fluorescence microscopy (Keyence BZ-9000).*
Experimental factors	*Immunohistochemistry was performed for CAR, S100β, and the stem/progenitor markers (SOX2, E-cadherin, Nestin, and Vimentin).*
Experimental features	*Sections were prepared from embryonic rat tissues fixed with 4% (w/v) paraformaldehyde. After immuno-reaction with primary antibodies, fluorescein isothiocyanate-, Cy3-, or Cy5-conjugated secondary antibodies were used for detection.*
Data source location	*Kawasaki, Kanagawa, Japan*
Data accessibility	*Data are within the present article*
Related research article	*M. Chen, T. Kato, M. Higuchi, S. Yoshida, H. Yako, N. Kanno, Y. Kato, Coxsackievirus and adenovirus receptor-positive cells compose the putative stem/progenitor cell niches in the marginal cell layer and parenchyma of the rat anterior pituitary,* Cell Tissue Res., 354, 2013, 823–836. http://www.ncbi.nlm.nih.gov/pubmed/24057874[1].

**Table:** 

**Value of the data** • This data could guide further investigation of maintenance and differentiation of the brain. • This data extend our understanding of the role of CAR in stem/progenitor cells in the embryonic brain and in the choroid plexus of the brain ventricle. • The data provide a basis for further explorations of the detailed role of CAR in the brain.

## 1 Data

**Fig. 1:**
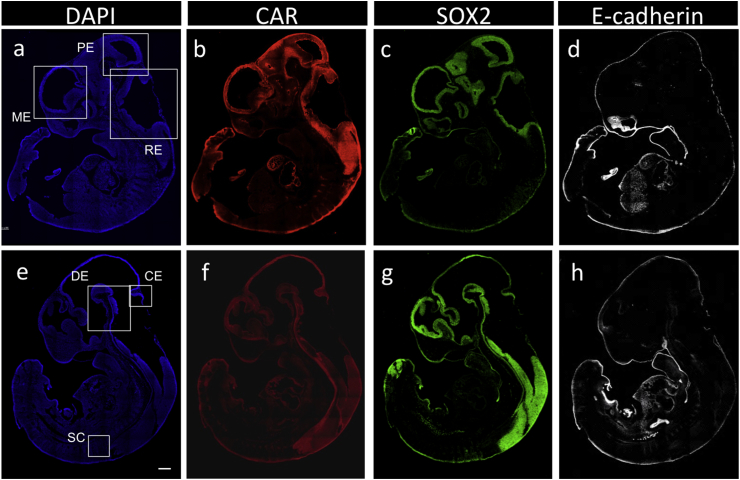
**Overall localization of CAR, SOX2, and E-cadherin in embryos on E13.5**. Nuclei staining with DAPI (*blue,***a** and **e**) and immunohistochemical analyses of CAR (Cy3, *red*, **b** and **f**), SOX2 (FITC, *green*, **c** and **g**), and E-cadherin (Cy5, *white*, **d** and **h**) were performed for lateral (**a**–**d**) and medial (**e**–**h**) regions. Boxed regions in **a** and **e** are enlarged in Fig. 2, Fig. 3. PE; prosencephalon, ME; mesencephalon, RE; rhombencephalon, DE; diencephalon, CE; cerebellum, SC; spinal cord. *Scale bar* 500 μm.

**Fig. 2:**
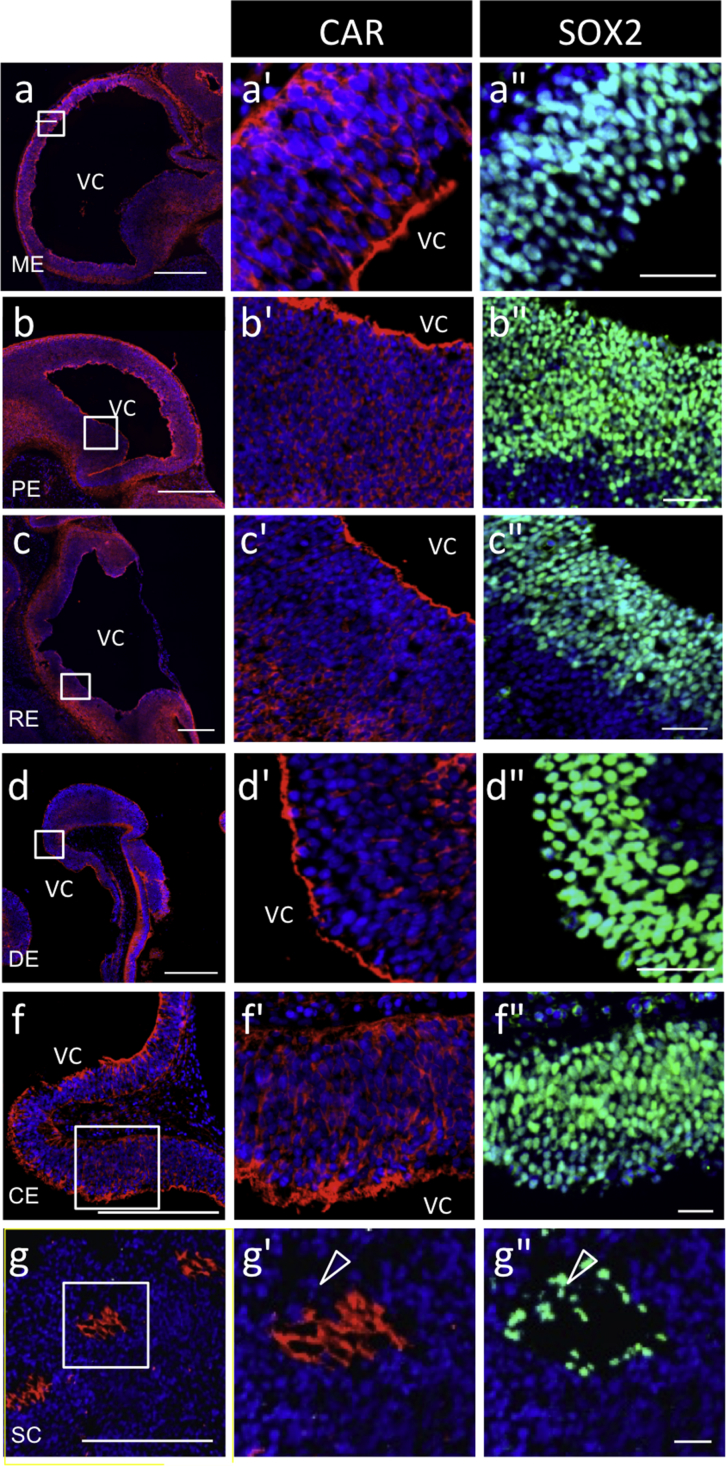
**Localization of CAR in embryos on E13.5**. Color codes are the same as those in Fig. 1. Each boxed area in **a**–**g** is enlarged in **a'**–**g'** (CAR) and **a''**–**g''** (SOX2) as merged images with DAPI. Open arrowheads indicate a SOX2-single positive cell in the ependymal zone of the spinal cord. VC; ventricular cavity, ME; mesencephalon, PE; prosencephalon, RE; rhombencephalon, DE; diencephalon, CE; cerebellum, SC; spinal cord. *Scale bars* 500 μm (**a**–**g)**, or 50 μm (**a''**–**g''**).

**Fig. 3:**
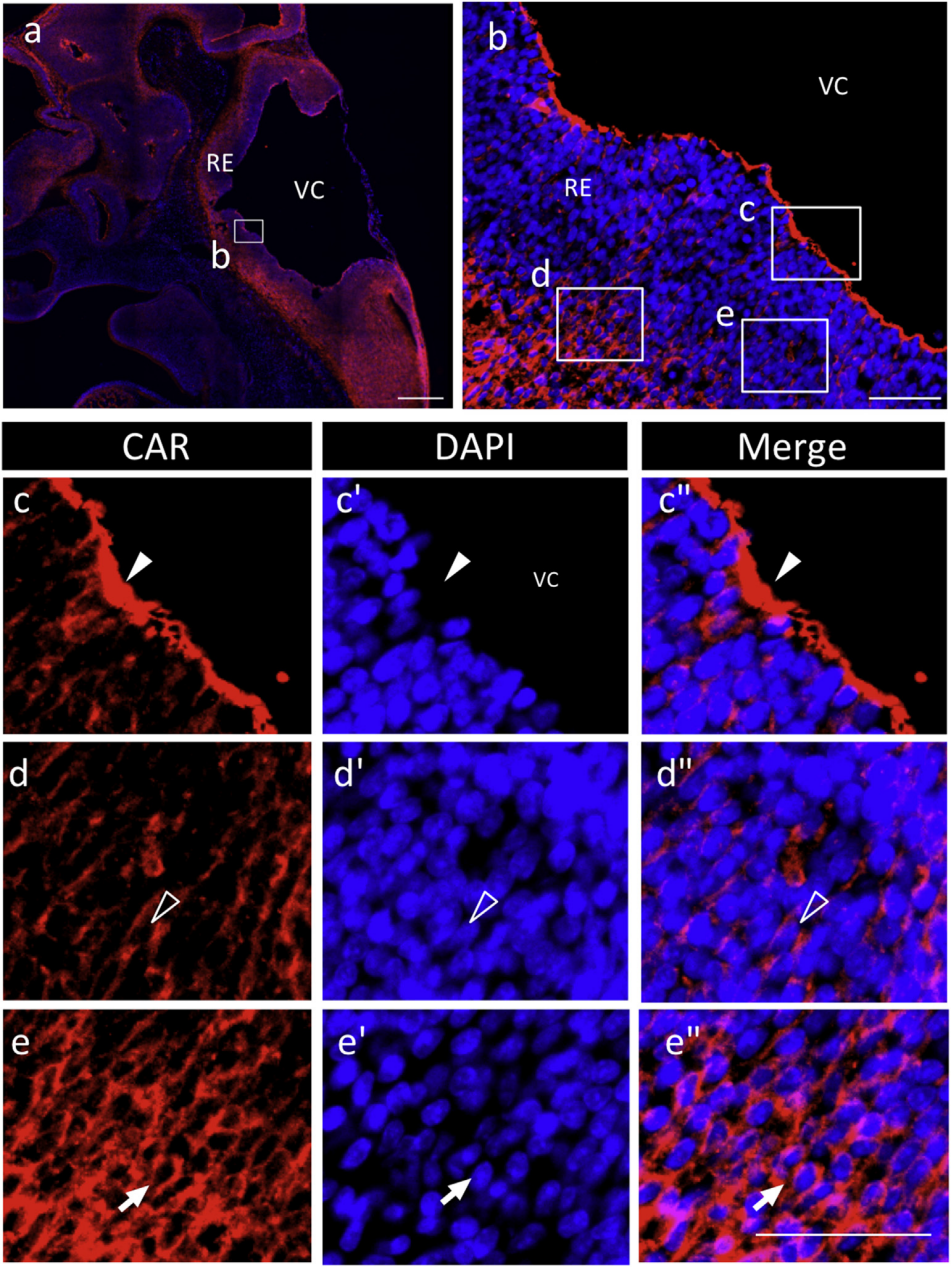
**Localization of CAR-positive cells in the rhombencephalon of embryos on E13.5**. Color codes are the same as those in Fig. 1. Boxed areas in **b**, part of the rhombencephalon (RE) of the rat embryo (boxed in **a**), are further enlarged (**c**–**c''**; ependymal zone/ventricular zone, **d**–**d''**; mantle zone/intermediate zone, **e**–**e''**; marginal zone). Arrowheads, open arrowheads, and arrows indicate the polarized localization of CAR; apical, basolateral, and round localization, respectively. VC; ventricular cavity. *Scale bars* 500 μm (**a)**, or 50 μm (**b**–**e''**).

Using whole bodies on embryonic day 13.5 (E13.5), immunohistochemical analyses showed extensive CAR, which plays multifold functions [1], [2], [3], [4], [5], [6], and SOX2 signals in the embryonic brain, while E-cadherin was mostly negative (Fig. 1). Enlarged merged images with DAPI showed the colocalization of CAR and SOX2, including the spinal cord (Fig. 2). Characteristic apical, basolateral, and pericellular localization of CAR in the hindbrain were observed (Fig. 3).

**Fig. 4:**
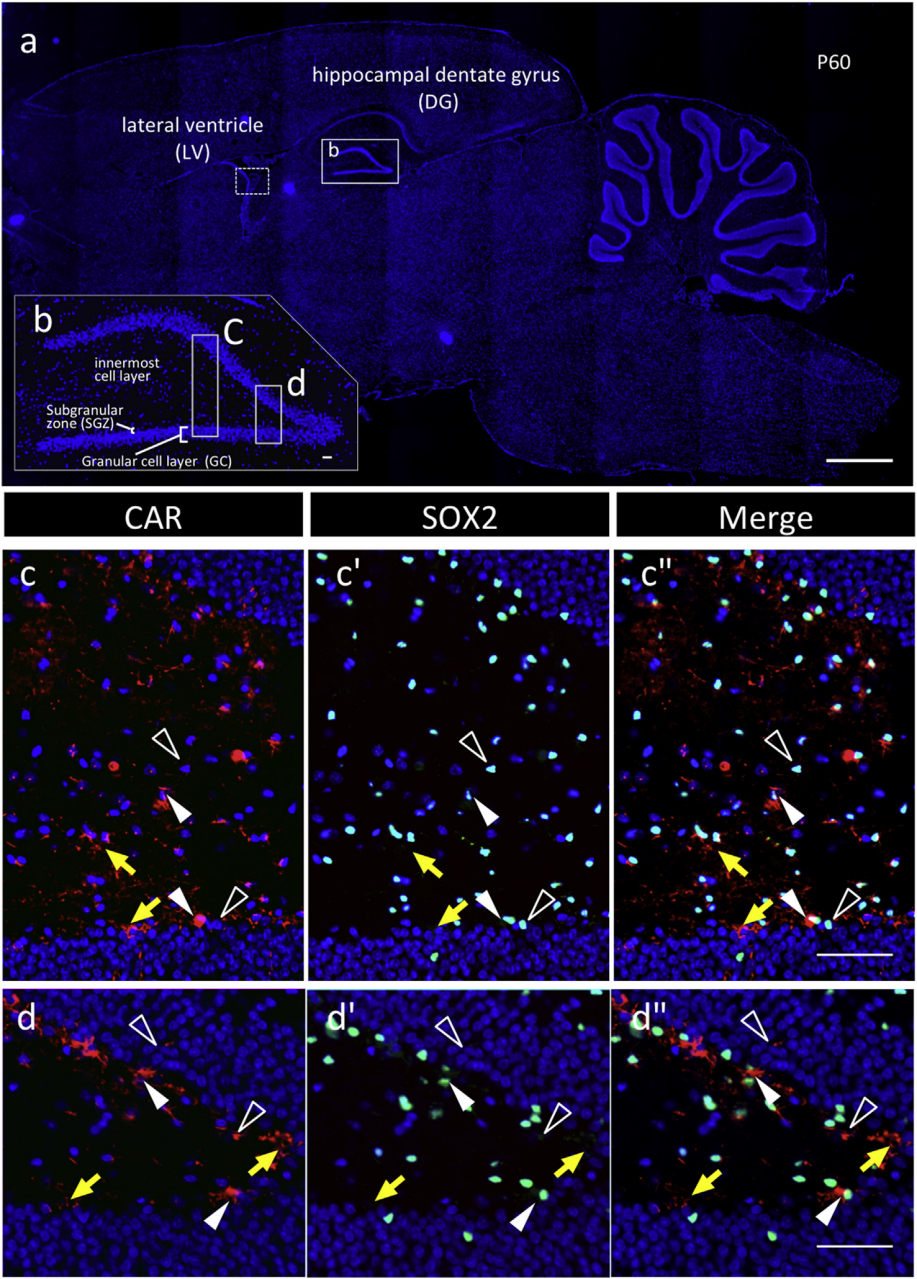
**Localization of CAR/SOX2-double positive cells in the hippocampal dentate gyrus of P60**. Color codes are the same as those in Fig. 1. In the sagittal section of the brain (**a**), boxed areas and dotted frames are the hippocampal dentate gyrus (DG) and lateral ventricle (LV) and are enlarged in **b** and in Fig. 6, respectively. Boxed areas in **b** are enlarged in **c**–**c''** and **d**–d**''**, respectively, with images of CAR and SOX2 as merged images with DAPI. Arrowheads and open arrowheads indicate CAR/SOX2-double and CAR-single positive cells, respectively. Yellow arrows indicate the polarized localization of CAR. *Scale bars* 500 μm (**a)**, or 50 μm (**b**–**d''**).

**Fig. 5:**
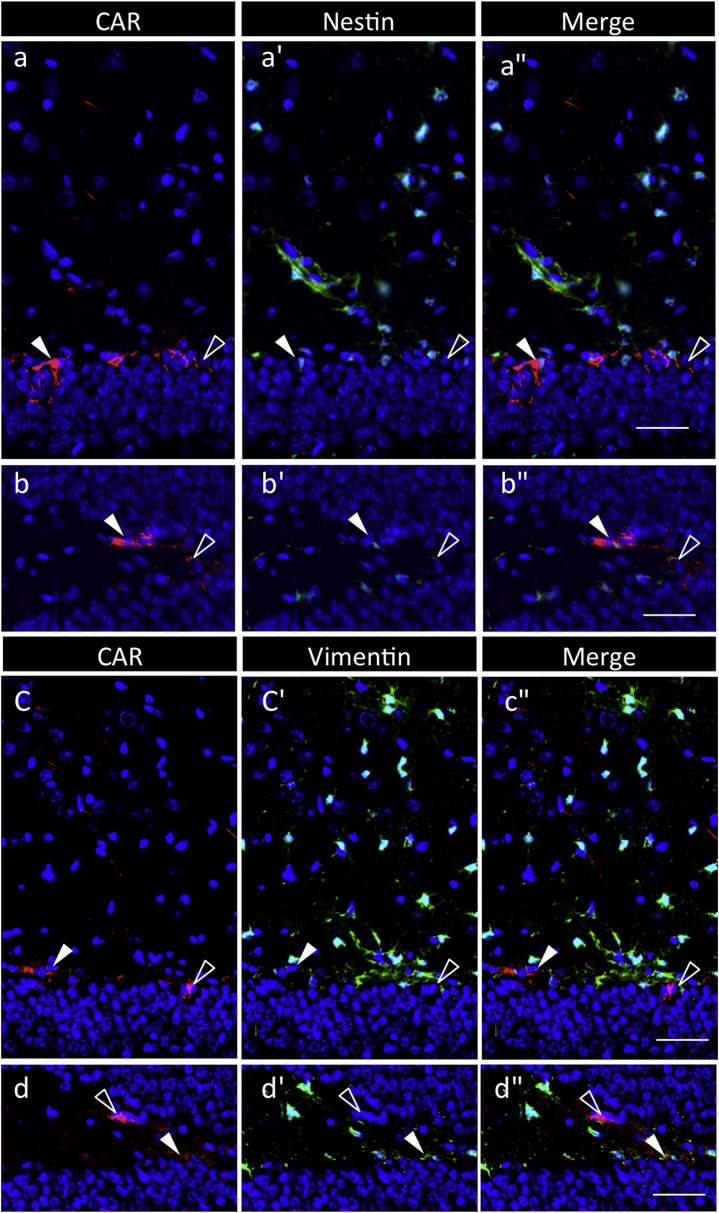
**Localization of CAR/Nestin-double or CAR/Vimentin-double positive cells in the hippocampal dentate gyrus of rat P60**. CAR (red)/Nestin (green)-double (**a**–**a**'', **b**–**b**'') or CAR/Vimentin (green)-double (**c**–**c**'', **d**–**d**'') positive cells in the rostral (**a**–**a**'', **c**–**c**'') and distal (**b**–**b**'', **d**–**d**'') areas of the hippocampal dentate gyrus are enlarged as merged images with DAPI. Arrowheads and open arrowheads indicate CAR/Nestin (or Vimentin)-double and CAR-single positive cells, respectively. *Scale bars* 50 μm.

**Fig. 6:**
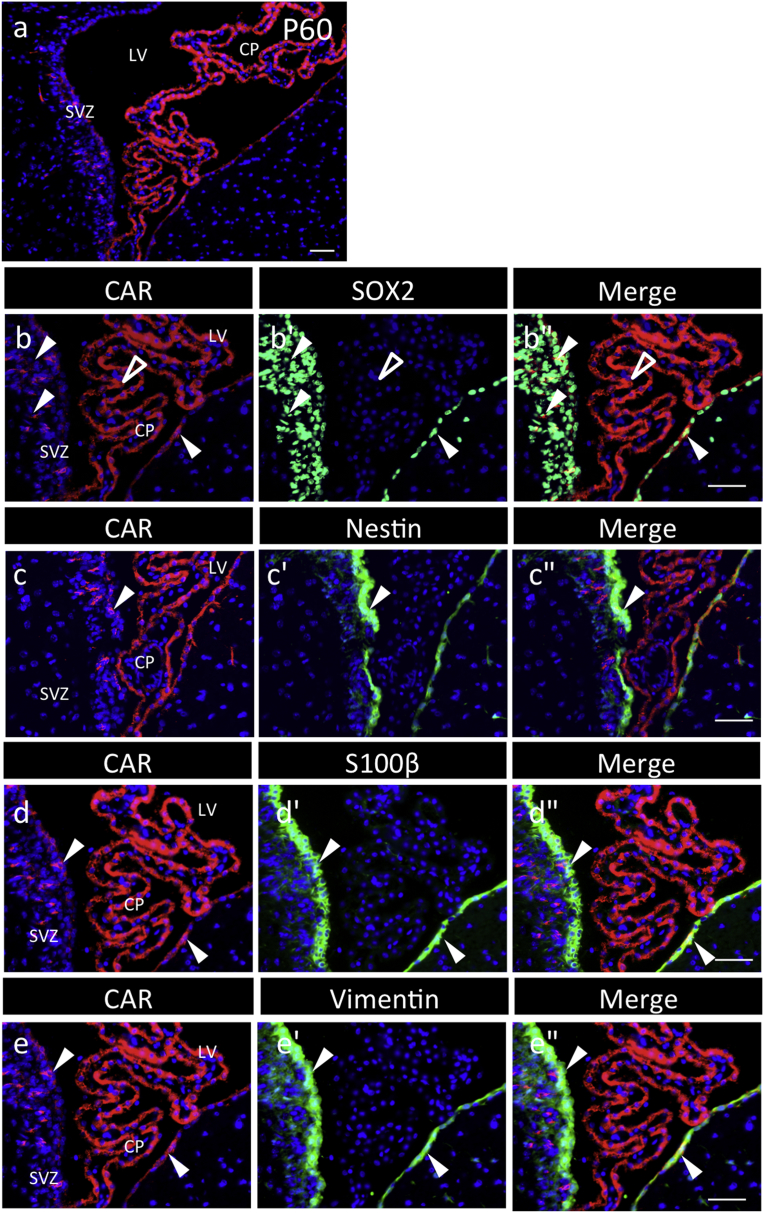
**Characterization of CAR-positive cells in the subventricular zone of rat P60**. Color code is the same as that in Fig. 5. CAR-positive cells in the subventricular zone (SVZ) were characterized by staining together with SOX2 (green, **b**–**b**''). Enlarged images (green) of Nestin (**c**–**c**''), S100β (**d**–**d**''), and Vimentin (**e**–**e**'') in part of the SVZ are shown as merged image of CAR and DAPI. Arrowheads and open arrowheads indicate CAR/SOX2 (Nestin, S100β or Vimentin)-double and CAR-single positive cells, respectively. SVZ; subventricular zone, LV; lateral ventricle, CP; choroid plexuses. *Scale bars* 50 μm.

Using the adult brain on postnatal day 60 (p60), CAR localization was examined in the subgranular zone (SGZ) of the hippocampus and the subventricular zone (SVZ), which are known as the stem/progenitor cell niche. CAR-positive signals were found in the SGZ and in the innermost cell layer (Fig. 4); some cells were also positive for Nestin and Vimentin (Fig. 5). In the SVZ, CAR signals were observed in SOX2-positive cells in the ependymal cell layer, but some negatives in those of the choroid plexus, and in multiple cell layers of the parenchyma bordering the ventricular cavity, with polarized localization (Fig. 6). CAR-positive cells of ependymal cells, except for those in the choroid plexus, were also positive for Nestin, S100β, and Vimentin (Fig. 6). Similar profiles were observed in the fourth ventricle (data not shown).

## 2 Experimental design, materials and methods

### 2.1 Animals

Wistar-crlj S100β-GFP transgenic strain rats, which express the reporter gene green fluorescent protein (GFP) under the control of the S100β-promoter [7], and Wistar-Imamichi strain rats were used. S100β-GFP transgenic rats were used only for analysis of P60. Breeding of rats and sampling of brains were described in the previous paper [1]. The present experimental design was approved by the Institutional Animal Care and Use Committee, Meiji University and was performed in accordance with the NIH Guidelines for the Care and Use of Laboratory Animals.

### 2.2 Immunohistochemistry

Procedures of an antigen retrieval, fixation and immunostaining were performed as described previously [1] using primary antibodies and secondary antibodies under the conditions listed in Table 1.

**Table 1:** 

A. List of primary antibodies				
Primary antibody	Species	Isotype	Working dilution	Vendor (Area)
CAR	Rabbit	IgG	1:500	Santa Cruz Biotechnology (Dallas, Tex., USA)
SOX2	Goat	IgG	1:400	Neuromics (Edina, Minn., USA)
GFP	Chicken	IgY	1:500	Aves labs (Tigard, Ore., USA)
E-cadherin	Mouse	IgG	1:200	BD Biosciences (San Jose, Calif., USA)
Nestin	Mouse	IgG	1:250	BD Bioscience
Vimentin	Mouse	IgG	1:10000	Sigma (Saint Louis, Mo., USA)

B. List of secondary antibodies				
Secondary antibody	Species/Isotype	Label	Working dilution	Vendor (Area)
Anti-rabbit IgG	Donkey/IgG	Cy3	1:500	Jackson ImmunoResearch (West Grove, Pa., USA)
Anti-goat IgG	Donkey/IgG	FITC	1:500	Jackson ImmunoResearch
Anti-chicken IgY	Donkey/IgG	FITC	1:500	Jackson ImmunoResearch
Anti-mouse IgG	Donkey/IgG	Cy5	1:400	Jackson ImmunoResearch
Anti-mouse IgG	Donkey/IgG	FITC	1:500	Jackson ImmunoResearch
